# Autophagy induction targeting mTORC1 enhances *Mycobacterium tuberculosis* replication in HIV co-infected human macrophages

**DOI:** 10.1038/srep28171

**Published:** 2016-06-15

**Authors:** Anna-Maria Andersson, Blanka Andersson, Christoffer Lorell, Johanna Raffetseder, Marie Larsson, Robert Blomgran

**Affiliations:** 1 Division of Medical Microbiology, Department of Clinical and Experimental Medicine, Faculty of Health Sciences, Linköping University, Linköping, Sweden; 2 Division of Molecular Virology, Department of Clinical and Experimental Medicine, Faculty of Health Sciences, Linköping University, Linköping, Sweden

## Abstract

To survive and replicate in macrophages *Mycobacterium tuberculosis* (Mtb) has developed strategies to subvert host defence mechanisms, including autophagy. Autophagy induction has the potential to clear Mtb, but little is known about its effect during controlled tuberculosis and HIV co-infection. Mammalian target of rapamycin complex1 (mTORC1) inhibitors were used to induce autophagy in human macrophages pre-infected with HIV-1_BaL_ and infected with a low dose of Mtb (co-infected), or single Mtb infected (single infected). The controlled Mtb infection was disrupted upon mTOR inhibition resulting in increased Mtb replication in a dose-dependent manner which was more pronounced during co-infection. The increased Mtb replication could be explained by the marked reduction in phagosome acidification upon mTOR inhibition. Autophagy stimulation targeting mTORC1 clearly induced a basal autophagy with flux that was unlinked to the subcellular environment of the Mtb vacuoles, which showed a concurrent suppression in acidification and maturation/flux. Overall our findings indicate that mTOR inhibition during Mtb or HIV/Mtb co-infection interferes with phagosomal maturation, thereby supporting mycobacterial growth during low-dose and controlled infection. Therefore pharmacological induction of autophagy through targeting of the canonical mTORC1-pathway should be handled with caution during controlled tuberculosis, since this could have serious consequences for patients with HIV/Mtb co-infection.

*Mycobacterium tuberculosis* (Mtb) is the causative agent of tuberculosis (TB), which in 2012 led to 1.3 million deaths. This disease is further fuelled by the rapid spread of drug-resistant TB strains and by HIV. HIV increases the risk of developing active TB thirtyfold thereby enhancing the mortality rates^1^. To avoid the resistance problem and the adverse interactions between the TB antibiotic rifampicin and anti-retroviral treatment against HIV^2^,^3^ it has been suggested that targeting or strengthening the immune response offer new treatment options against TB^4^,^5^,^6^. Evidence points towards autophagy as being an essential component in the immune response against TB and its modulation can thereby act as a potential therapeutic target^6^. Rapamycin-induced autophagy in mice has been shown to enhance the efficacy of the BCG vaccine by increasing the antigen presentation in dendritic cells^7^. Efforts made to improve this using DNA vaccines targeting autophagy have also shown efficacy^5^,^8^. However, inhibiting mammalian target of rapamycin (mTOR) to induce autophagy as a mean to treat Mtb infected macrophages has mostly been studied for short periods of infection^9^,^10^, focusing on Mtb viability before the bacterium has had the chance to undergo one replication cycle. It is important, however, to understand how autophagy induction influences Mtb replication during latent infection and HIV co-infection. HIV/Mtb co-infection is a challenge to treat^11^, since these pathogens demonstrate synergistic effects upon co-infection^12^, contributing to the increased mortality.

Autophagy is not only a way for the cell to gain nutrients by degrading cellular components during starvation, but is also an important defence mechanism against intracellular pathogens^13^,^14^. Autophagy can be triggered or enhanced by vitamin D3^15^,^16^, TLR-mediated signalling during phagocytosis^17^,^18^,^19^,^20^, cellular starvation, or by inhibition of mTOR by rapamycin or Torin1^21^. Torin1 is an ATP-competitive inhibitor of mTORC1 that is a more specific blocker than the allosteric inhibitor rapamycin^21^. Characteristic of autophagy is the formation of a double membrane surrounding a target, creating an autophagosome by the aid of autophagy related (ATG) proteins^13^,^22^. The canonical autophagy marker, the microtubule-associated protein 1 light chain 3 beta (LC3B; ATG8) attaches in its lipidated form (LC3 II) to the autophagosomal membrane and interacts with sequestosome 1 (SQSTM1: also known as p62), which delivers polyubiquitinated protein aggregates and bacteria to the autophagosome^13^,^14^,^23^,^24^. Upon maturation, autophagosomes fuse with lysosomes forming autophagolysosomes where the captured target is degraded^13^,^14^. In order to survive intracellularly HIV and Mtb have developed several strategies^9^,^25^,^26^. The process by which HIV modulates autophagy is complicated as HIV inhibits autophagy, by decreasing the number of autophagosomes^27^, as well as utilizing autophagy for its replication^28^. This may be explained by the different stages in HIV infection, with short term infections making the virus more vulnerable to autophagy induction^27^, while the virus in long term infections has established a way to utilize early autophagy processes in its favour. At this stage the HIV protein Nef is able to inhibit the later stages with autophagosome maturation through interactions with the autophagy regulatory factor Beclin1^28^.

Many studies have shown that viable and metabolically active Mtb inhibits phagolysosome fusion in order to survive, whereas heat-killed and dormant bacteria have a high level of phagosome-lysosome fusion^9^,^25^,^26^,^29^,^30^. Nevertheless, blocking of IL-10 or inducing autophagy through starvation or the mTOR inhibitor rapamycin can overcome this inhibition and promote phagosomal maturation, leading to decreased survival of Mtb^9^,^31^. Autophagy induction through mTORC1 inhibition limits both HIV infection^27^ and Mtb infection^9^. Nevertheless, less is known about their combined or synergistic effect during latent TB infection, and whether pharmacological induction of autophagy can be a therapeutic strategy to overpower Mtb also in HIV/Mtb co-infected macrophages.

This study was designed to investigate the role of autophagy induction on phagosome maturation and Mtb replication in Mtb and HIV/Mtb co-infected human monocyte derived macrophages (hMDMs). Since latency is the most common outcome of Mtb infection in humans^32^, we set up an *in vitro* system with a low dose of Mtb (MOI = 1 or lower) which enable human macrophages to control the infection for at least 7–8 days, in contrast to a higher MOI of 10 when Mtb replicates uncontrolled^33^,^34^. We found that Mtb and HIV/Mtb co-infection triggered autophagy although inhibited phagosomal maturation. Pharmacological autophagy induction interfered with the control of Mtb in low dose infected hMDMs, causing a further suppression in phagosomal maturation and Mtb to replicate. This was especially pronounced in HIV co-infected cells.

## Results

### HIV/Mtb co-infection increases LC3B puncta formation and association of LC3B to Mtb phagosomes

**Figure 1:**
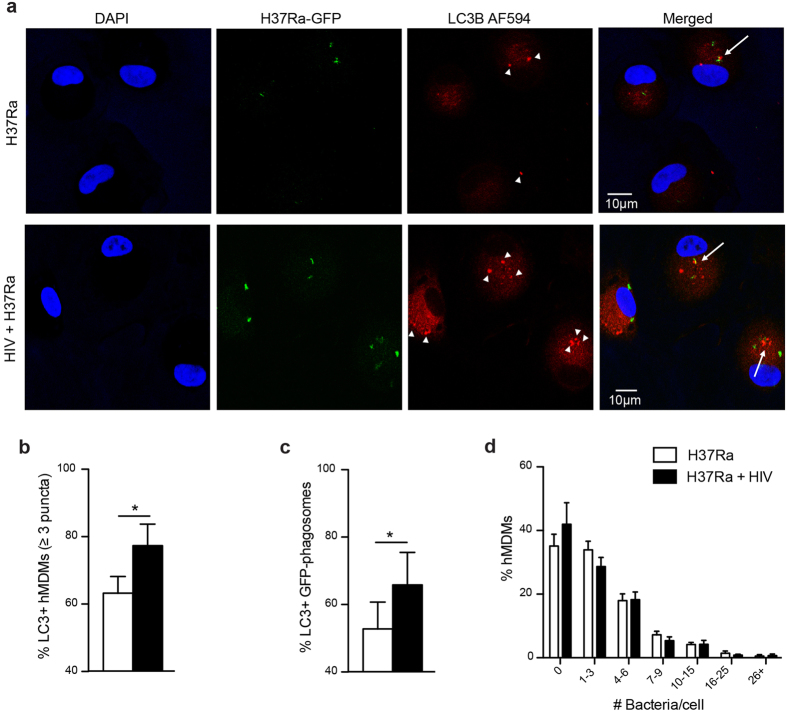
HIV/Mtb co-infection increases LC3B puncta formation and association of LC3B to Mtb phagosomes. (**a**) Representative micrographs of LC3B puncta formation (arrowheads) and co-localization to Mtb phagosomes (arrows) in hMDMs infected with HIV (7 days) and/or H37Ra (MOI = 1, 2 h). Blue: DAPI, green: Mtb, red: LC3B (AlexaFluor594). (**b**) Percentage of LC3B positive hMDMs (3 puncta or more). (**c**) Percentage of LC3B positive Mtb phagosomes. (**d**) Percentage of hMDMs infected with the indicated number of bacteria/cell, showing no difference in Mtb infection with or without HIV. Data are mean ± SEM from 10 independent experiments in which 50–100 phagosomes were counted for each condition. *p < 0.05 using paired Student t-test.

Autophagy in Mtb or HIV/Mtb co-infected hMDMs was evaluated by analysing LC3B puncta formation, a characteristic of autophagosomes. Cells infected with HIV for seven days were infected for 2 h with Mtb H37Ra-GFP before they were analysed for formation of puncta as well as the puncta co-localization to Mtb phagosomes (Fig. 1a–c). Co-infection significantly increased the frequency of LC3B+ hMDMs, compared to that of single infected cells (p = 0.037) (Fig. 1a,b). Evaluating LC3B+ vs. LC3B- Mtb-phagosomes further showed an increased association of LC3B puncta to phagosomes in co-infected hMDMs (p = 0.029 for single vs. co-infected) (Fig. 1a,c). This is in line with previous findings, where HIV triggered autophagy by increased expression of LC3 II after several days of infection in hMDMs^28^. The increased puncta formation and co-localization to Mtb phagosomes could not be explained by a higher infection burden in co-infected cells, since there were no differences in the number of bacteria in single and co-infected cells (Fig. 1d).

### Rapamycin accelerates H37Ra/H37Rv replication in both single and HIV co-infected hMDMs

**Figure 2:**
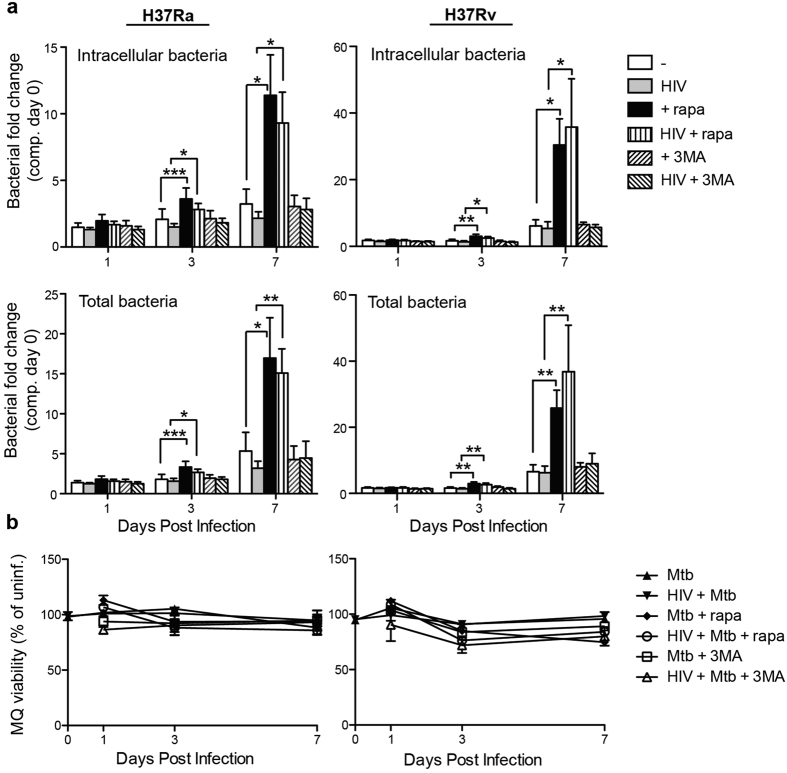
mTOR inhibition using rapamycin accelerates H37Ra/H37Rv replication in both single and HIV co-infected hMDMs. (**a**) hMDMs were pre-infected with/without HIV for three days before infection with H37Ra or H37Rv (MOI = 1) for 2 hours. Rapamycin (rapa; 1 μM) or 3-MA (1 mM) was added for 1, 3, and 7 days and the signal from luciferase expressing H37Ra or H37Rv in cell lysates and supernatant were measured. “Total bacteria” is the combined supernatant + intracellular pool of bacteria. (**b**) At the indicated time-points hMDMs (MQ) viability compared to uninfected hMDMs was measured using calcein AM uptake. Data are mean ± SEM from 6 independent experiments. *p < 0.05, **p < 0.01, ***p < 0.001 using paired Student t-test.

Next, we investigated the effect of rapamycin-induced autophagy in HIV/Mtb co-infected cells, using a low MOI of Mtb. Induction of autophagy has been shown to decrease the survival of Mtb through phagolysosomal maturation^9^. However, the intracellular Mtb replication significantly increased upon stimulation with rapamycin, evident at day 3 and further increased at day 7, for both the avirulent (H37Ra) and the virulent (H37Rv) Mtb strain (Fig. 2a). At day 7, H37Rv increased from 6.1-fold to 30.4-fold (p = 0.012) in single infected and from 5.4-fold to 35.8-fold (p = 0.031) in HIV/H37Rv co-infected cells, without or with rapamycin treatment, respectively. Inhibition of autophagy by 3-MA on the other hand had no effect on Mtb replication in either single or co-infected hMDMs. Assessing total amount of bacteria, i.e. amounts found in cell lysate and supernatant (Fig. 2a, second panel), did not further increase the bacterial yield compared to cell lysate alone (Fig. 2a, top panel), indicating that rapamycin affected Mtb growth inside of hMDMs. In order to exclude that the differences seen were due to cell death the viability of hMDMs was measured, showing no differences over the course of infection compared to uninfected cells (Fig. 2b). Rapamycin (and 3-MA) did not have any effect on Mtb growth in absence of hMDMs (Supplementary Fig. S1), further illustrating that rapamycin affects Mtb intracellular growth by manipulating the hMDMs and not by targeting the bacteria directly.

### Torin1 inhibits phosphorylation of mTORC1 downstream targets and induces autophagy more efficiently than rapamycin

**Figure 3:**
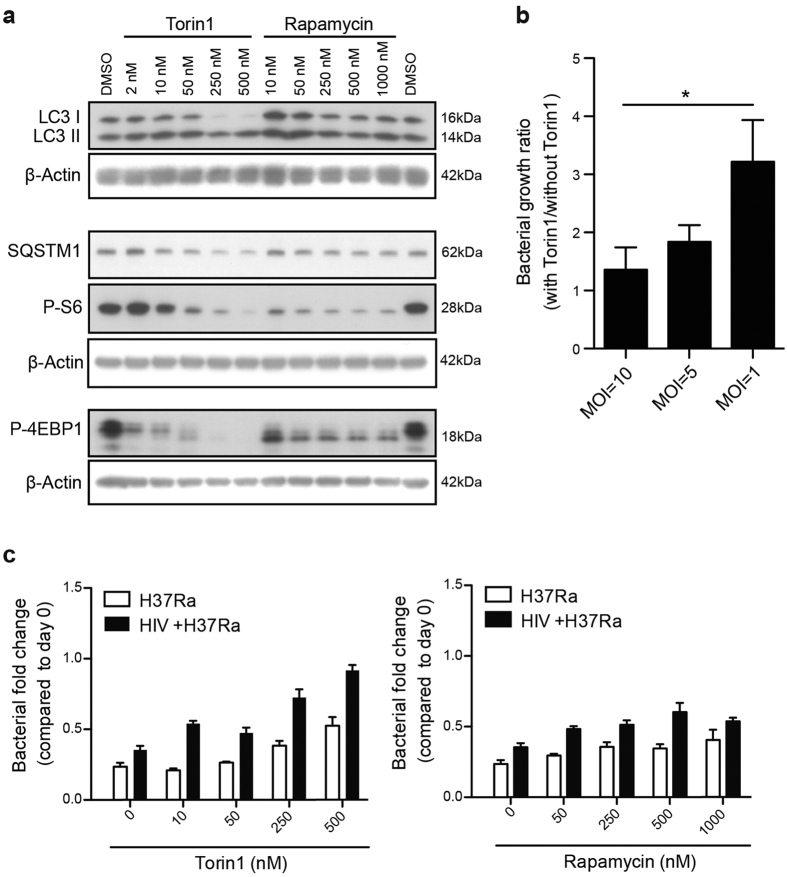
The efficient mTORC1 inhibitor Torin1 causes a dose-dependent increase in Mtb growth in co-infected hMDMs at low MOI. (**a**) Representative immunoblots from two independent experiments showing the dose-response of Torin1 and rapamycin on the autophagy markers LC3B and SQSTM1 (p62) and phosphorylation of the mTORC1 downstream targets S6 and 4EBP1, with their respective β-actin loading controls, after 6 h treatment. Full length of the cropped blots are shown in Supplementary Fig. S3. (**b**) hMDMs were infected at the indicated MOI for 2 h, and then treated with/without 250 nM Torin1 for 3 days. The signal from luciferase expressing H37Rv was quantified, and the graph shows the ratio in total bacteria (lysate + supernatants) for Torin1 treated vs. untreated. Data are mean ± SEM from 6 independent experiments. *p < 0.05 using paired Student t-test. (**c**) hMDMs were pre-infected with/without HIV for seven days before infected with H37Ra (MOI = 0.2) for 2 hours. hMDMs were then incubated with/without rapamycin or Torin1 at increasing concentrations for 3 days. Graphs show the level of intracellular bacteria in cell lysates compared to day 0. Data are mean ± SEM from triplicate, representative of two independent experiments.

Rapamycin enhanced Mtb growth in our system, which is in accordance with findings by Zullo *et al*.^35^. We therefore further investigated how effective rapamycin was at inducing autophagy. To this end we compared rapamycin to another autophagy inducer, Torin1, which is a more selective and potent ATP-competitive mTORC1 inhibitor than rapamycin^21^. Autophagy was evaluated by assessing the protein expression levels of the autophagy markers LC3 and SQSTM1 (also known as p62), and indirectly by assessing the inhibition of the mTORC1 pathway, as detected by decreased phosphorylation of its downstream targets S6 and 4EBP1. Torin1 was more efficient in inducing autophagy and autophagic flux compared to rapamycin (Fig. 3a). This was demonstrated by more concentration dependent de-phosphorylation of S6 and 4EBP1, as well as conversion of LC3 I to LC3 II, together with degradation of the autophagy substrate SQSTM1 (Fig. 3a).

### In a controlled infection, mTOR inhibition causes a dose-dependent increase in Mtb growth, especially in HIV co-infected hMDMs

After confirming that Torin1 was a more potent mTORC1 inhibitor and autophagy inducer in hMDMs than rapamycin, we investigated the impact of Torin1 on macrophages infected at various doses of Mtb. With decreasing dose of Mtb, autophagy induction had a greater impact on bacterial growth (Fig. 3b), indicating a higher sensitivity towards autophagy induction when infection is controlled. To examine if there were differences in the sensitivity of Mtb infected versus HIV/Mtb co-infected cells to autophagy induction, macrophages infected with a low dose of Mtb were treated with increasing concentrations of the autophagy inducers. There was a pronounced control of Mtb infection at day 3, which was lost in HIV/Mtb co-infected hMDMs with rapamycin and to higher extent when stimulated with Torin1 (Fig. 3c).

### Torin1 increases Mtb replication independently of the time of stimulation

**Figure 4:**
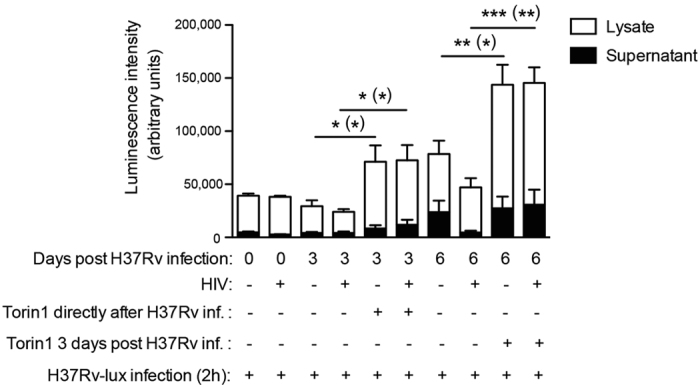
Same effect of early and late autophagy induction in Mtb infected hMDMs. hMDMs were pre-infected with/without HIV for seven days before infected with H37Rv (MOI = 1) for 2 hours. hMDMs were then incubated with/without Torin1 (250 nM) for 3 days, added either directly or 3 days post infection. The graph shows the level of bacteria in cell lysates and supernatants. Data are mean ± SEM from 5 independent experiments. *p < 0.05, **p < 0.01, ***p < 0.001 were * represent the significance for lysate while (*) represents the significance for total bacteria (lysate + supernatant) using paired Student t-test. (See also Supplementary Fig. S2 for CFU confirmation of these lysates).

To see if changing the timing of autophagy induction also affected Mtb replication in hMDMs, we stimulated autophagy using Torin1 either directly after phagocytosis or 3 days post infection. Torin1 caused a significant increase in Mtb replication both in Mtb single (p = 0.023) and HIV/Mtb co-infected (p = 0.029) hMDMs when mTORC1 inhibition was started 2 h after infection and was maintained for the 3 first days of infection (Fig. 4). More importantly, we found that even with an established infection that was controlled at day 3, Torin1 still caused an extensive increase in Mtb replication to 6 days post infection (Fig. 4). The difference in bacterial fold between Torin1 treated and untreated at day 6 was more pronounced in HIV/Mtb co-infected hMDMs (1.67-fold increase in single infected and 3.41-fold increase in co-infected). The amount of bacteria was measured both in the lysate and in the supernatant showing that the majority of the bacteria resided inside the cells. Luciferase data of lysate was also confirmed by CFU plating of the same lysates, showing the same results (Supplementary Fig. S2).

### HIV/Mtb co-infection inhibits phagosomal fusion with lysosomes, which is further decreased upon mTORC1 inhibition

**Figure 5:**
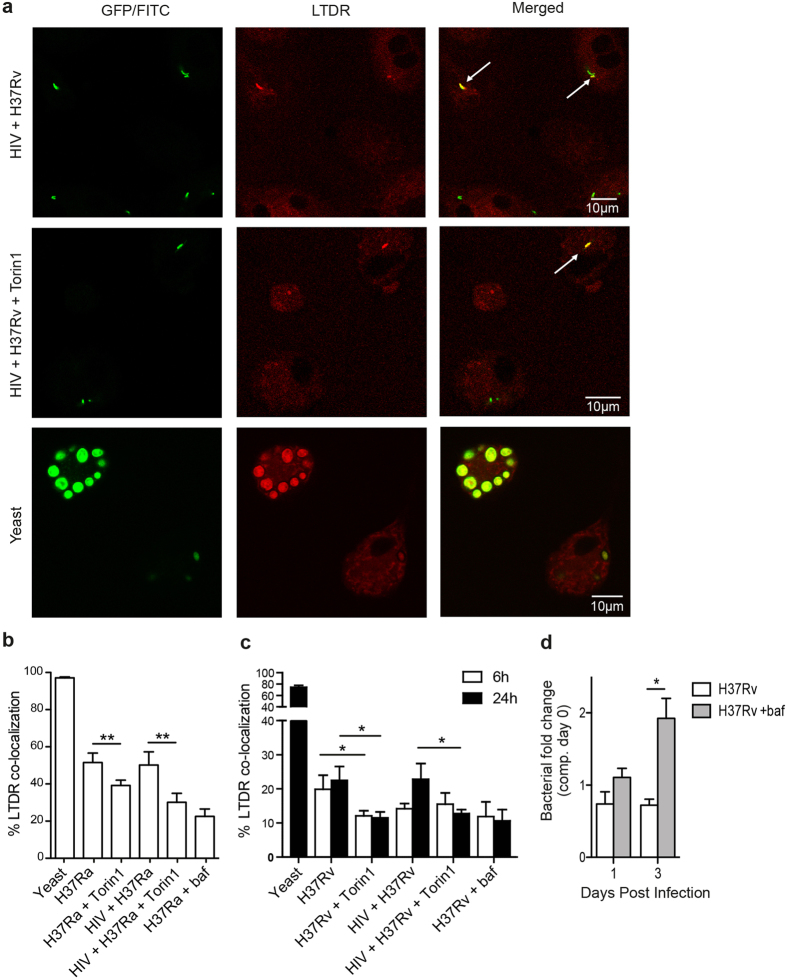
HIV/Mtb co-infection inhibits phagosomal fusion with lysosomes, which is further decreased upon autophagy induction. (**a**) Representative micrographs of LysoTracker Deep Red (LTDR) co-localization to phagosomes in hMDMs infected with yeast (MOI = 5) or co-infected with HIV/H37Rv for 6 h (7 days pre-infection with HIV), unstimulated and stimulated with Torin1 (250 nM) the last 4 h. The arrows in the HIV/H37Rv co-infected micrographs indicate co-localization to some of the Mtb phagosomes. All yeast particles in the lower micrographs exhibited co-localization. Green: Mtb or yeast, red: LTDR, yellow: co-localization. (**b**) Percentage of LTDR co-localization with yeast or H37Ra (MOI = 1) phagosomes 6 h post infection. (**c**) Percentage of LTDR co-localization with H37Rv (MOI = 1) phagosomes 6 and 24 h post infection. (**d**) hMDMs were pretreated with bafilomycin (baf; 100 nM) for 1h before infection with luciferase-expressing H37Rv (MOI = 1) for 2 h. Extracellular bacteria were washed away, and baf was re-added every 12 h. The combined luminescence signal from supernatant and hMDM-lysate (=total bacteria) are shown. Data are mean ± SEM with *p < 0.05 and **p < 0.01 using paired Student t-test, of 3 independent experiment for (**d**) and six independent experiments for LysoTracker data at 6 h and eight independent experiments for 24 h (n = 6 for yeast) in which 100–200 phagosomes were counted for each condition.

Since mycobacterial killing is subsequent to phagosomal maturation^36^ and increased killing has been correlated with increased acidification^9^, we hypothesized that the decreased killing of Mtb during autophagy induction was due to a decreased phagosomal acidification. In order to study maturation of Mtb phagosomes and how HIV affects this process, LysoTracker Deep Red co-localization was assessed. This probe stains organelles with low pH such as lysosomes^37^. HIV pre-infected hMDMs were infected with Mtb for 6 h or 24 h before co-localization of phagosomes with LysoTracker was analysed. The positive phagolysosome control, yeast, showed a near to complete co-localization of LysoTracker (97%) after 6 h whereas co-localization was greatly reduced for Mtb with approximately 50% for both single and HIV/H37Ra co-infected cells (Fig. 5b) and a further reduction in H37Rv infected/co-infected cells at both 6 h and 24 h (Fig. 5a,c).

Although Torin1-treatment had no effect on yeast phagosome maturation (Supplementary Fig. S4a), autophagy induction caused an additional 10% decrease in phagolysosome fusion in H37Ra infected hMDMs (exhibiting 40% co-localization with LysoTracker; p = 0.002) and a 20% decrease in co-infected cells (exhibiting 30% co-localization with LysoTracker; p = 0.004), similar to the levels of the negative control bafilomycin (Fig. 5b). CD63 co-localization was analysed in addition to LysoTracker co-localization and revealed a Mtb/CD63 co-localization that was similar to that of the bafilomycin control (~15%) (Supplementary Fig. S4b). mTORC1 inhibition of Mtb-single or HIV co-infected hMDMs did not further improve this co-localization (Supplementary Fig. S4b). Torin1-reduced phagolysosome fusion, e.g. LysoTracker co-localization, in H37Rv infected hMDMs was similar to that in H37Ra. At 6 h H37Rv infected hMDMs exhibited a 1.7-fold increase to bafilomycin (p = 0.006), while co-infected cells showed no increase (1.02-fold increase; p = 0.95 compared to bafilomycin), indicating that HIV co-infection by itself inhibits phagosomal maturation. To monitor differences in LysoTracker co-localization with Torin1 stimulation also during HIV co-infection with H37Rv, phagosomal maturation were additionally analysed at 24 h. At this time point Torin1 significantly inhibited LysoTracker co-localization in both single (p = 0.025) and co-infected (p = 0.035) hMDMs (Fig. 5c). In both the uninfected and H37Rv infected cells, mTOR inhibition did not affect the total protein levels of proteins involved in phagosome/autophagosome maturation, i.e. levels of LAMP-1 and CD63 were unchanged (Supplementary Fig. S4c,d). These results reveal that autophagy induction further impair the already deficient phagosomal maturation in HIV co-infected hMDMs. Such a decrease in phagosomal maturation/acidification can reduce the control of Mtb^33^ and we found that bafilomycin treatment had this effect as the hMDMs ability to control replication of H37Rv decreased and gave a more than 2.6-fold higher bacterial load compared to untreated cells day 3 post infection (p = 0.02) (Fig. 5d). These data would explain the increased sensitivity towards mTOR inhibition that results in accelerated Mtb growth particularly in HIV co-infected cells (as seen in Fig. 3c, and with day 3 to 6 treatment in Fig. 4).

### Mtb inhibits autophagic flux, and Torin1 treatment leads to a further build-up of the autophagic substrate SQSTM1

**Figure 6:**
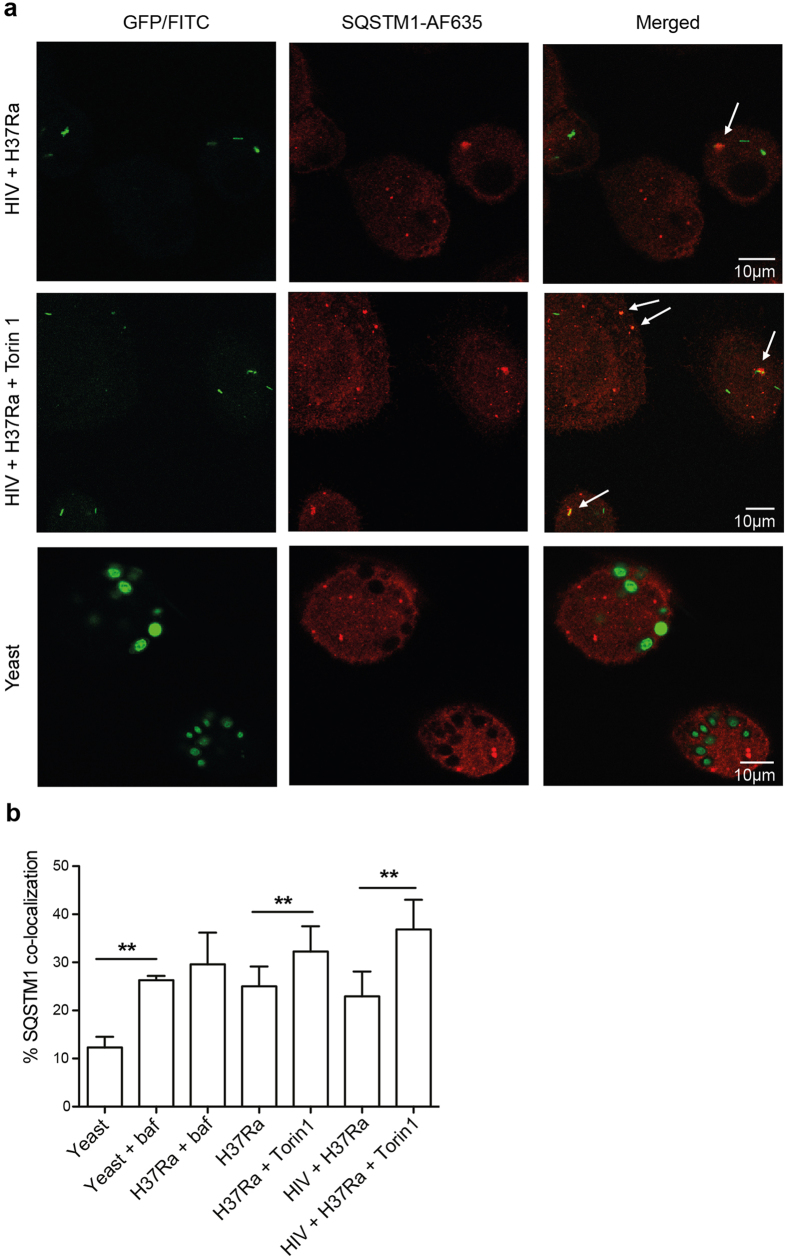
Mtb inhibits autophagic flux, and autophagy induction cause further build-up of SQSTM1 in Mtb phagosomes. (**a**) Representative micrographs of SQSTM1 recruitment/accumulation to phagosomes in hMDMs infected with yeast (MOI = 5) or co-infected with HIV and Mtb (MOI = 1) for 6 h (7 days pre-infection with HIV), unstimulated and stimulated with Torin1 (250 nM) the last 4 h. Some cells were treated with bafilomycin (baf; 100 nM) 1 h prior to Mtb/yeast infection. The arrows indicate SQSTM1 co-localization to Mtb or yeast phagosomes. Green: Mtb or yeast, red: SQSTM1. (**b**) Percentage of SQSTM1 co-localization with yeast or H37Ra phagosomes. Data are mean ± SEM with **p < 0.01 using paired Student t-test, of six independent experiments (n = 3 for yeast+baf) in which 100–300 phagosomes were counted for each condition.

Autophagic flux was then analysed, focusing on the degradation or accumulation of the autophagy substrate SQSTM1, which delivers polyubiquitinated protein aggregates and bacteria to the autophagosome^13^,^14^,^23^,^24^. SQSTM1 co-localization was evaluated by confocal microscopy in infected or co-infected macrophages. Phagosomes in hMDMs containing yeast for 6 h had a low level of SQSTM1 co-localization (~10%; Fig. 6a,b), unless cells were pre-treated with bafilomycin which increased the co-localization more than 2-fold (p = 0.004; Fig. 6b). Bafilomycin was used to prevent degradation of SQSTM1 in case of lysosomal fusion leading to autophagic flux, and reveals an active flux in macrophages infected with yeast. These differences with and without bafilomycin treatment was not seen in macrophages infected with Mtb, indicating that Mtb indeed inhibits the autophagic flux (Fig. 6b). Similar degree of SQSTM1 co-localization between yeast+ bafilomycin-treated hMDMs and Mtb infected hMDMs indicates that autophagy is present in both samples, although Mtb inhibits the autophagic flux while yeast does not. Furthermore, Torin1 increased SQSTM1 positive phagosomes with 1.3-fold in the single Mtb infected hMDMs (p = 0.006), and with 1.6-fold in the HIV co-infected hMDMs (p = 0.003). This is in agreement with LysoTracker data, indicating that Torin1 further inhibits acidification and maturation of Mtb-phagosomes causing reduced autophagic flux with build-up of SQSTM1, which is more pronounced in HIV co-infected hMDMs. Furthermore, LC3-LysoTracker-Mtb co-localization studies indicated that the level of maturing autophagosomes, e.g. LC3+LysoTracker+Mtb+ autophagolysosomes, were lower in HIV co-infected than in Mtb-single infected hMDMs. In addition, mTORC1 inhibition increased the number of autophagosomes (LC3+Mtb+), without increasing the number of autophagolysosomes (Supplementary Fig. S5). Although Torin1 induced autophagy and flux on a cellular level (whole cell lysates of uninfected hMDMs; Fig. 3a), we found that when the cells are infected with Mtb, there is still a decreased flux on the subcellular level, localized specifically to the Mtb phagosomes.

### Torin1-induced autophagy and flux is cellular and not localized to Mtb phagosomes

**Figure 7:**
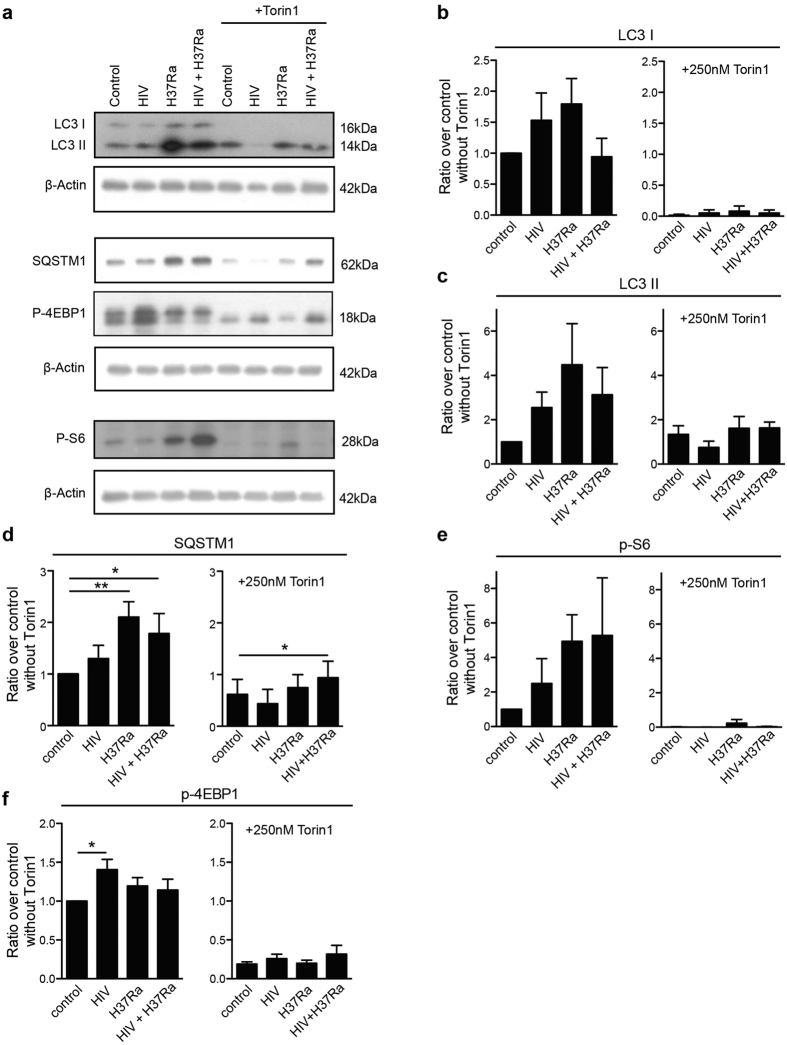
Torin1-induced autophagy and flux is cellular and not localized to Mtb phagosomes . (**a**) Representative immunoblots from seven independent experiments showing the autophagy markers LC3B and SQSTM1 (p62) and the phosphorylation of the mTORC1 downstream targets S6 and 4EBP1, with their respective β-actin loading controls. The hMDMs were pre-infected for seven days with HIV before 6 h Mtb infection (MOI = 1), with the addition of Torin1 (250 nM) the last 4 h. Full length of the cropped blots are shown in Supplementary Fig. S6. (**b–f**) Densitometry measurements normalized to their respective β-actin control and presented as ratio over control without Torin1, shown as mean ± SEM with *p < 0.05 and **p < 0.01 using repeated measurement ANOVA comparing all treatments against its control (n = 7).

After analysing autophagy and flux on a subcellular level (Figs 1,6, and Fig. S5) we further analysed these processes in single and HIV/Mtb co-infected cells at a cellular level. Analysing the total protein expression, we observed an induction of autophagy with inhibited flux in Mtb and co-infected cells (Fig. 7), seen by the increased conversion of LC3 I into LC3 II and the accumulation of the autophagy substrate SQSTM1. When adding Torin1, autophagy was induced in both single and co-infected cells, as seen by the complete conversion of LC3 I into LC3 II, which together with SQSTM1 had decreased considerably indicating autophagic flux. Similarly, autophagy induction in dendritic cells could also overcome the cellular Mtb-inhibited flux^38^. However, co-infected hMDMs stimulated with Torin1 retained an increased level of SQSTM1 (p = 0.042) compared to its Torin1 stimulated control (Fig. 7a,d). In order to further evaluate if HIV manipulates autophagy in co-infected cells, autophagic flux was assessed using the flux inhibitor bafilomycin. For both SQSTM1 and LC3 II, there was no cellular flux (baf/without baf = 1 or less) in Mtb and HIV/Mtb co-infected hMDMs in absence of Torin1. Torin1 stimulation on the other hand increased the cellular flux of both SQSTM1 (p = 0.029) and LC3II (p = 0.047) in Mtb infected but not in HIV/Mtb co-infected hMDMs (Supplementary Fig. S7a,b). Furthermore, HIV infected hMDMs displayed enhanced 4EBP1 phosphorylation, as well as partial resistance to de-phosphorylation by Torin1 in HIV only and HIV co-infected hMDMs (Fig. 7a,f). The decreased phosphorylation of the mTORC1 targets S6 and 4EBP1 by Torin1 reflects the initiation of early steps in the autophagy process, consistent with a previous study^39^. In the absence of Torin1 this was counteracted by the pathogens in both single and co-infected hMDMs (Fig. 7a,e,f), although Mtb only increased S6 phosphorylation without affecting 4EBP1, as also seen previously^40^.

By making a distinction between overall cellular autophagy and subcellular autophagy, our findings indicate that Torin1-induced flux in hMDMs only affects the overall cellular autophagy, but do not overcome the flux inhibited by Mtb inside the phagosome. As shown in previous figures, this process was especially accentuated in phagosomes of HIV co-infected hMDMs leading to increased replication of Mtb.

### HIV modulated expression of essential *ATG* genes during co-infection

**Figure 8:**
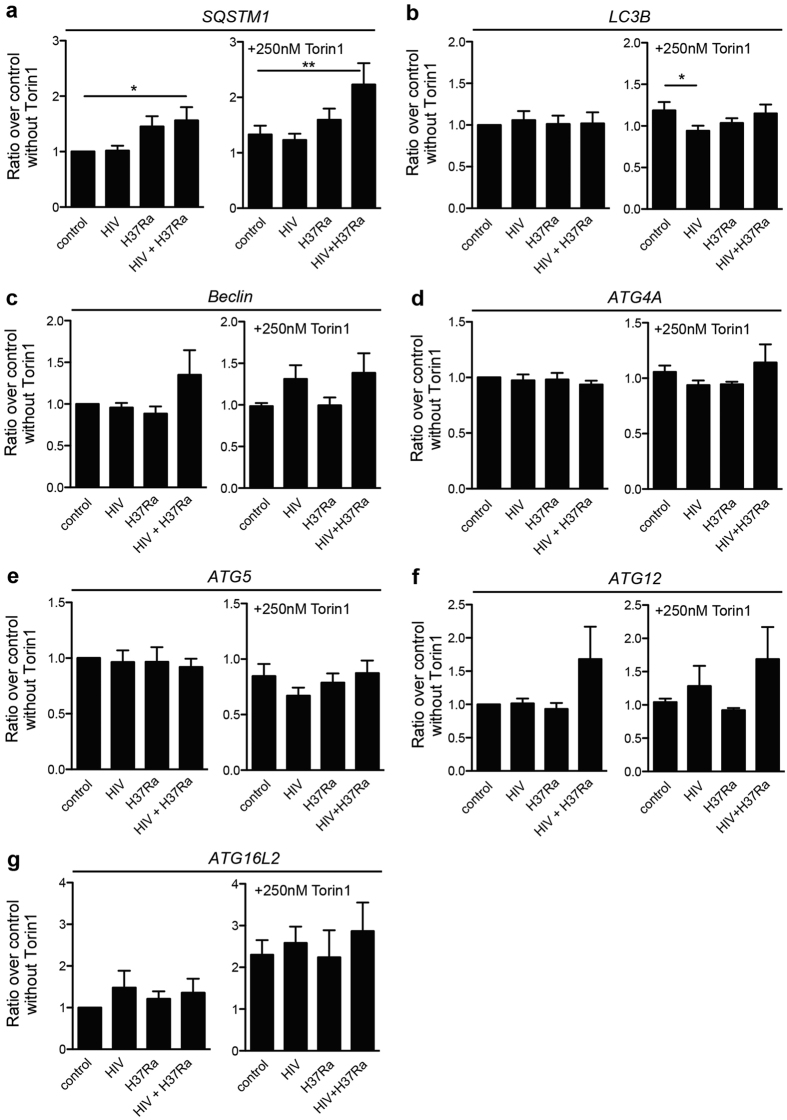
HIV modulated expression of essential *ATG* genes during co-infection. hMDMs were pre-infected with/without HIV for seven days before 6 h Mtb infection (MOI = 1), adding Torin1 (250 nM) the last 4 h. Trizol was added to the infected and stimulated hMDMs to extract RNA. RNA was extracted from the same samples as analyzed for protein expression in Fig. 7. The gene expression were analyzed for: (**a**) *SQSTM1*, (**b**) *LC3B*, (**c**) *Beclin1*, (**d**) *ATG4A*, (**e**) *ATG5*, (**f**) *ATG12*, and (**g**) *ATG16L2*. The changes in gene expression are presented as ratios over control without Torin1, shown as mean ± SEM with *p < 0.05 and **p < 0.01 using repeated measures ANOVA (n = 5–6).

Gene expression analyses were performed to evaluate differential expression of *ATG* genes, and whether the decrease in autophagy proteins during Torin1 treatment correlates with a lowered gene expression. Similar to the protein levels, *SQSTM1* showed an increased gene expression upon co-infection (Fig. 8a), but in contrary to the protein levels gene expression analysis revealed that *SQSTM1* was unaffected or even increased with Torin1 treatment (Fig. 8a). In contrast to the evident differences of LC3B (ATG8) at protein level (Fig. 7a–c) there were no significant differences in *LC3B* gene expression levels in the absence of Torin1 (Fig. 8b). Upon Torin1 treatment, HIV pre-infected hMDMs resisted the modest increase in *LC3B* expression seen in the uninfected control (p = 0.0303). *Beclin1/ATG6, ATG4A, 5, 12*, and *ATG16L2* have previously shown to be differentially expressed during infection with the intracellular pathogen *Francisella tularensis*^41^. Although no significant changes were detected for these genes (Fig. 8c–g), there was a noteworthy increase in *Beclin1/ATG6* and *ATG12* expression during co-infection, which was not seen in Mtb single infected hMDMs even when stimulated with Torin1 (Fig. 8c,f).

Together the gene expression analyses indicates that some *ATG genes* (*Beclin1/ATG6* and *ATG12*) could be differentially expressed during co-infection, but that the degradation of LC3B and SQSTM1 proteins caused by Torin1 do not correlate with decreased gene expression of these autophagy markers, but instead is due to an overall increased cellular autophagic flux.

## Discussion

Although utilizing autophagy induction as a strategy in the development of future vaccines against TB shows promise^5^,^8^, autophagy induction as treatment of latently TB infected individuals could be a different scenario altogether. Using a low grade Mtb infection where human macrophages are able to keep Mtb in check for at least 7 days^33^, we herein describe the loss of this control with inhibition of phagosomal maturation upon mTORC1 inhibition during HIV co-infection.

We observed an increase in both amount of LC3 positive cells and LC3 positive phagosomes in HIV co-infected hMDMs compared to Mtb single infected, indicating a pathogen-enhanced induction of autophagy. When this process was induced pharmacologically by inhibition of mTORC1 it broke the cellular control of Mtb and caused an increased Mtb replication particularly in the HIV co-infected cells. One explanation for this enhanced replication is the augmented inhibition in phagolysosome fusion during autophagy induction. Collectively, these results indicate that mTORC1 inhibition can have a favourable effect for Mtb, by disrupting the ‘balanced control’ that together with HIV causes an uncontrolled growth of Mtb. Similar results were observed in HIV infected cells, in which rapamycin stimulation resulted in a higher viral load^28^. HIV infection also accumulated LC3 due to inhibition of autophagic maturation^28^. In addition, other studies have proved that knockdown of several *ATG* genes or inhibition of autophagy with 3-MA lowers HIV replication^42^,^43^. Autophagy also increased the replication of *Salmonella*, while knockdown of *ATG* genes decreased it^44^. Clearly these findings indicate that autophagy can be favourable for the intracellularly adapted pathogens. Studying human primary macrophages much of the observed effects were at the level of protein modifications, such as increased phosphorylation of the mTORC1 targets S6 and 4EBP1, LC3 I – LC3 II conversion, and suppressed autophagic flux. These data suggest that Mtb modulates autophagy in several ways: i) by recruiting pre-existing LC3, ii) activating mTORC1 thereby suppressing further autophagophore formation, and iii) by preventing degradation of formed autophagosomes/phagosomes. HIV enhances the Mtb-induced modulation of autophagy, as indicated by the lack of cellular autophagic flux even with a potent autophagy inducer such as Torin1. During Torin1 treatment HIV co-infected cells also maintained a low frequency of mature autophagosomes (e.g. autophagolysosomes, LC3+LysoTracker+Mtb+) compared to autophagosomes (LC3+Mtb+), thereby supporting the intracellular survival of Mtb. Furthermore, the slight increased expression of *Beclin1* and *ATG12* could also explain the early autophagosome formation which might make co-infected cells more sensitive to mTORC1 inhibition resulting in accentuated suppression of phagosome acidification and flux inhibition.

Upon autophagy stimulation with Torin1, we observed a decrease in the overall cellular level of autophagy proteins, indicating degradation and an active flux. This cellular flux was not inhibited by the pathogens, which mostly had its inhibiting effect localized to the phagosomes in which Mtb resided. This was seen subcellularly by the decreased phagosome maturation and further accumulation of SQSTM1 by Torin1, especially pronounced in co-infected hMDMs. This observation reveals the importance of analysing subcellular compartments rather than the cell as a whole. Contrary to Mtb, phagocytosis of yeast, resulted in increased co-localization with LysoTracker and a decreased co-localization to SQSTM1 due to degradation of this protein upon acidification, as indicated by rescue experiment using bafilomycin. Mtb-infected hMDMs, however, showed no difference in SQSTM1 co-localization with or without bafilomycin, indicating absence of SQSTM1 degradation and inhibition of the autophagic flux. Petruccioli *et al*.^45^ also demonstrated that Mtb inhibited the autophagic flux, but that this inhibition could be overcome by Mtb-specific T cells, ultimately increasing killing of the bacteria^45^. However, in the absence of T-cells, we showed that co-infected hMDMs were especially sensitive to autophagy induction by Torin1 and rapamycin, resulting in an uncontrolled intra-macrophage growth of Mtb. The increase in Mtb growth in HIV co-infected cells has previously been shown by Imperiali *et al*.^46^, who demonstrated a further increase in Mtb replication upon stimulation with TNF-α and IFN-γ, which was only evident in HIV co-infected cells^46^. Since these cytokines are known to induce autophagy^10^,^47^ it is possible that induction of autophagy in these co-infected cells was the reason behind the increased Mtb replication.

In contrast to our findings, BCG or Mtb infected RAW 264.7 cells stimulated with high doses of rapamycin decreased bacterial viability^9^, in addition to inducing maturation of BCG and *M. smegmatis* phagosomes^9^,^40^. Zullo *et al*. showed that higher doses of rapamycin or Torin1 induce killing of *M. smegmatis* independently of autophagy. In addition, this group also showed that low doses of rapamycin or Torin1 was not sufficient to induce killing of mycobacteria in murine macrophages although it was enough to reduce mTOR signalling and induce autophagy^35^, indicating that autophagy induction and killing of mycobacteria is uncoupled in murine macrophages. In our study low doses of rapamycin and Torin1 efficiently inhibited mTORC1 and induced autophagy in hMDMs, but at the same time stimulated an increased replication of Mtb. Besides differences in concentrations of autophagy stimuli, we show that the effects of autophagy induction depend on the bacterial dose and whether the macrophage is controlling the infection or not. Here we have used a low MOI to observe a controlled TB infection, and upon mTOR inhibition we have seen a loss of control of Mtb growth. During infection with high bacterial burden, autophagy induction might be beneficial, but during a low dose infection with Mtb our data clearly indicate that mTOR inhibition disturbs the controlled growth. This increased Mtb proliferation was more pronounced in HIV co-infected cells compared to Mtb single infected hMDMs, as seen clearly when lowering the bacterial MOI. More studies are needed to elucidate if the disturbance of the phagosome, or whether or not the access to nutrients, could be the reason behind the increased Mtb replication in autophagy-induced HIV co-infected hMDMs detected herein. Although we have found a negative role for pharmacological autophagy induction on Mtb killing using a low dose of Mtb, it is possible that inducing autophagy by other means can provide a better protection against this infection.

In conclusion our study shows that in human macrophages HIV and Mtb co-infection induces autophagy but have an inhibiting effect on phagosomal maturation. Upon autophagy induction with the mTOR inhibitor Torin1 there was a further suppression in phagosomal maturation, together with an increased formation of autophagosomes (SQSTM1 and LC3 co-localization) that did not mature into autophagolysosomes, indicating that mTORC1 inhibition indeed locally reduces autophagic flux. Co-infected hMDMs were more sensitive to pharmacological autophagy induction, resulting in a lost control over Mtb in human macrophages. We have found that mTORC1 inhibition can disrupt the growth equilibrium of Mtb in infected cells and trigger an increase in Mtb replication, which most likely is caused by the decreased phagosomal acidification seen upon mTORC1 inhibition. Until proven otherwise we suggest that autophagy induction as a treatment option for tuberculosis should be handled with caution since co-infection with HIV could increase the replication of Mtb and in an *in vivo* situation possibly activate TB in latently infected individuals.

## Materials and Methods

### Generation of macrophages from monocytes

Monocytes were isolated from buffy coats (Linköping blood bank) from healthy donors who had given written consent for research use of the donated blood in accordance with the Declaration of Helsinki, not requiring a specific ethical approval according to paragraph 4 of Swedish law (2003:460) on Ethical Conduct in Human Research. Buffy coats were mixed with 0.9% NaCl and carefully added on top of Lymphoprep (Axis-Shield PoC AS). The tubes were centrifuged (450 × g, 40 min, 20 °C) and isolated monocyte bands were washed with PBS-heparin and with Krebs Ringer Glucose (KRG) without Ca^2+^ three times each before adhesion to tissue culture flasks for 90 min at 37 °C with 5% CO_2_. Non-adherent cells were removed by vigorous washing and the monocytes were cultured in DMEM supplemented with 10% pooled natural human serum (Linköping blood bank) for seven days to mature into macrophages with medium replacement every 3–4 days.

### Preparation of *Mycobacterium tuberculosis*

*Mycobacterium tuberculosis* (Mtb) H37Ra or H37Rv was thawed and cultured in Mtb medium (Middlebrook 7H9 with 0.05% Tween-80, 0.5% glycerol and 10% ADC enrichment) supplemented with 20 μg/ml kanamycin for GFP-expressing Mtb or 100 μg/ml hygromycin for luciferase-expressing Mtb for two weeks and passaged one week before use. Log phase Mtb was pelleted (5,000 × g, 5 min) and separated by needle shearing first in PBS-Tween-80 (0.05%) and then in serum-free DMEM medium. The OD value was measured to determine bacterial concentration followed by calculation of multiplicity of infection (MOI), and verified by serial dilution and plating on 7H11 agar.

### HIV-1BaL

HIV-1BaL (Lot p4238) was produced using chronically infected cultures of the ACVP/BCP cell line (no. 204). Virus was purified and concentrated as previously described^48^ and aliquots were frozen in liquid nitrogen vapor.

### Co-infection with HIV and *Mycobacterium tuberculosis*

Human monocyte derived macrophages (hMDMs) were infected with 0.06 ng/ml HIV-BaL for three or seven days before used in experiments. At the day of experiment some cells were pre-treated with 100 nM bafilomycin A1 (from *Streptomyces griseus*, Sigma Aldrich) 1 h at 37 °C, 5% CO_2_ prior infection with live Mtb or with FITC labelled *Saccharomyces cerevisiae* (referred to as yeast; as positive stimuli showing high level of phagosomal maturation) at MOI = 5–10. After infection, the cells were incubated for different time points, depending on experiment.

### LysoTracker staining

LysoTracker Deep Red (Life technologies, Cat. No. L12492) was used to visualize acidic organelles. Infection of hMDMs with Mtb or yeast was followed by a 2 h incubation to allow phagocytosis before 250 nM of the selective mTOR inhibitor Torin1 (Tocris bioscience) was added to induce autophagy. Two or 22 hours after adding Torin1, LysoTracker was added to the final concentration of 75 nM and additionally incubated 2 h, in total making it 6 or 24 h of infection. hMDMs were washed with KRG and fixed with 4% paraformaldehyde (PFA). Cell contours were visualized by staining with wheat germ agglutinin (WGA) Alexa Fluor 350 (Life technologies) for 20 seconds, followed by mounting.

### Immunohistochemistry of SQSTM1, LC3 and CD63

hMDMs were infected with Mtb for 6 h, with/without 250 nM Torin1 the last 4 h, and stained for SQSTM1 or CD63 after fixation and WGA staining. Other hMDMs were stained for LC3 after Mtb infection for 2 h or for 6 h in case of co-staining with LysoTracker. The staining was performed as described previously^16^, using the primary antibodies monoclonal mouse anti-p62 (SQSTM1 D-3 Cat. No. sc-28359, Santa Cruz Biotechnology) diluted 1:800 and monoclonal mouse anti-LC3 (Code No. M152-3 Clone 4E12, MBL Medical & Biological laboratories Co) diluted 1:400. The secondary antibodies were Alexa Fluor 635 goat anti-mouse IgG (Life technologies) for SQSTM1, Alexa Fluor 594 Goat-anti-mouse IgG (Life technologies) for LC3 single staining and Alexa Fluor 568 goat anti-mouse IgG (Molecular probe) for LC3 and LysoTracker co-staining, all diluted 1:400. LC3 single stained cells were incubated in 1:100 diluted DAPI (Sigma Aldrich) 15 min before washing and mounting.

For CD63 staining hMDMs were permeabilized with 0.1% saponin (Sigma Aldrich) in 2% BSA and 10% goat serum (Dako, Denmark) for 30 min in RT. After washing, the monoclonal mouse antibody anti-CD63 (PeliCluster M1544, Sanguin, Amsterdam) diluted 1:50 was added for 1 h in RT. Secondary antibody Alexa Fluor 635 goat anti-mouse IgG (Molecular Probes, A31574) was added after washing, and incubated for 30 min at 37 °C before being fixed again, washed and mounted.

All cover slips were analysed in a LSM 700 Zeiss inverted confocal microscope with a plan-apochromat 40x, NA 1.3 objective to analyse LC3 positive cells, while the plan apochromat 63x, NA 1.40 objective was used for analysing co-localization to phagosomes. Images were acquired with Zen software and all samples were observed in a blinded-fashion and 50–300 phagosomes/sample were examined. Image brightness and contrast were adjusted equally with Photoshop for visualization of representative micrographs only, and this was done only after the completion of the blinded analysis.

### Mtb replication assay and cell viability

Triplicates of hMDMs that were pre-infected with HIV for 3 or 7 days or were pre-treated with bafilomycin for 1 h, were infected with H37Ra or H37Rv expressing luciferase for 2 h at the indicated MOI. After washing away extracellular bacteria, hMDMs were incubated with/without bafilomycin or the autophagy inhibitor 3-MA (1 mM) (Cat. No. M9281, Sigma) or the inducers rapamycin (Cat. No. R8781, Sigma) or Torin1 at the indicated concentrations and duration specified in figure legends. For measuring cell viability calcein-AM uptake was used after removal of supernatants. hMDMs were washed with PBS three times before 30 min incubation with 0.4% calcein (diluted in PBS). Fluorescence was measured in a Modulus microplate reader. After removing calcein the bacteria in both supernatants and lysates were measured as described previously^49^. Shortly, water was added to lyse the cells and after scraping they were transferred to a white modulus plate with water. After automatized addition of 1% of the substrate decanal, the luminescence from live bacteria in supernatant and lysate was quantified. For confirmation, CFU-determination was performed by serial dilutions and plating on 7H11 agar, enumerating colonies 2–3 weeks later.

### Protein and RNA isolation and RT-PCR

Cells grown in 12-well plates were infected and treated with/without the addition of 250 nM Torin1 as described above, and collected by adding 0.5 ml Trizol (Life technologies) 6 h after Mtb infection. Proteins and RNA were extracted according to the Trizol protocol. Briefly, after centrifugation, the upper aqueous phase was collected for RNA isolation and the rest was stored for protein isolation. Precipitated proteins were dried at 50 °C and re-suspended in 200 μl boiling 2x Laemmli sample buffer and were boiled for 10 min and sonicated for 5 min. The RNA was washed and treated to remove possible DNA contamination using TurboDNAse kit (Ambion) according to the manufacturer’s protocol. cDNA synthesis was performed using Superscript II enzyme (Cat. No. 18064-071, Life Technologies) according to manufacturer’s protocol and cDNA was purified using PureLink PCR Purification kit (Life Technologies).

Specific primers for selected genes were designed using the Primer3 program (http://frodo.wi.mit.edu/primer3/) and are shown in supplementary information.

Quantitative real-time PCR was performed in a 7900HT Fast Real-Time PCR (Applied Biosystems). One reaction contained 12.5 μl SYBR Green PCR Master Mix (Applied Biosystems), 1 μl of each primer (final concentration 800 nM) and 5 μl of cDNA. Total volume was adjusted with double distilled H_2_O to 25 μl. Three housekeeping genes (*β-actin*, *GAPDH* and *β2-microglobulin*) were used for normalization and the normalization factor was calculated from geometric mean of their Ct values^50^. The changes in gene expression are presented as ratios between hMDMs treated with stimuli and hMDMs without treatment.

### Western blot

hMDMs for western blot were collected by adding boiling 2x Laemmli sample buffer. Protein samples were re-boiled before separated on a tris glycine gel; Pager Gold Precast polyacrylamide (8–16%) gel (Lonza). Proteins were immobilized onto PVDF membranes Immobilon-P Transfer Membrane (Millipore), were blocked with 5% drymilk in PBS-Tween-20 (0.075%) for 1 h RT and probed with primary antibodies at 4 °C overnight. The antibodies were: rabbit monoclonal anti-LC3B (D11) (Cat. No. 3868, Cell signaling), mouse monoclonal anti-SQSTM1 D-3 (Cat. No. sc-28359, Santa Cruz Biotechnology), rabbit monoclonal anti-phospho-4EBP1 (Thr-37/46)(236B4) (Cat. No. 2855, Cell Signaling Technology), mouse monoclonal anti-p-Ribosomal Protein S6 Antibody (50.Ser 235/236, Cat. No. sc-293144, Santa Cruz Biotechnology), mouse monoclonal anti-human CD63 (clone H5C6, BD Biosciences), mouse monoclonal anti-LAMP-1 antibody (clone H4A3, Cat. No. sc-20011, Santa Cruz Biotechnology), and mouse monoclonal anti-β-actin (clone AC-74, Cat. No. A2228, Sigma Aldrich). The dilutions of the antibodies were 1:5,000 for LC3, 1:1,000 for SQSTM1 CD63 and LAMP-1, 1:2,000 for P-4EBP1 and P-S6, and 1:10,000 for β-actin.

The secondary antibodies polyclonal goat anti-rabbit or anti-mouse immunoglobulins/HRP (Dako Cytomation) were incubated for 1 h RT before developing, using ECL (Amersham pharmacia biotech). All secondary antibodies were diluted to 1:2,000 except for the one against anti-β-actin which had the dilution 1:10,000. Band intensities were quantified using ImageJ.

### Statistical analysis

All statistical analyses were performed with GraphPad prism software. The data were analyzed using paired Student t-test for comparison of two groups and repeated measurement ANOVA for multiple comparisons. p-values < 0.05 were considered significant.

## Additional Information

**How to cite this article**: Andersson, A-M. *et al*. Autophagy induction targeting mTORC1 enhances *Mycobacterium tuberculosis* replication in HIV co-infected human macrophages. *Sci. Rep.* **6**, 28171; doi: 10.1038/srep28171 (2016).

## Supplementary Material

Supplementary Information
