# Supplementary material for: Autophagy induction targeting mTORC1 enhances Mycobacterium tuberculosis replication in HIV co-infected human macrophages
Source: Sci Rep. 2016 Jun 15;6:28171. doi: 10.1038/srep28171 (PMC4908603; doi:10.1038/srep28171)
Supplement: Supplementary Information [file srep28171-s1.pdf]

Supplementary Information

Autophagy induction targeting mTORC1 enhances *Mycobacterium tuberculosis* replication in HIV co-infected human macrophages

Anna-Maria Andersson[1], Blanka Andersson[1], Christoffer Lorell[1], Johanna Raffetseder[1], Marie Larsson[2], and Robert Blomgran[1]*

[1]Division of Medical Microbiology, [2]Division of Molecular Virology, Department of Clinical and Experimental Medicine, Faculty of Health Sciences, Linköping University, Linköping, Sweden.

* To whom Correspondence should be addressed:

E-mail: robert.blomgran@liu.se (RB)

Phone: (+46) 10-1034778

Fax: (+46) 10-1034789

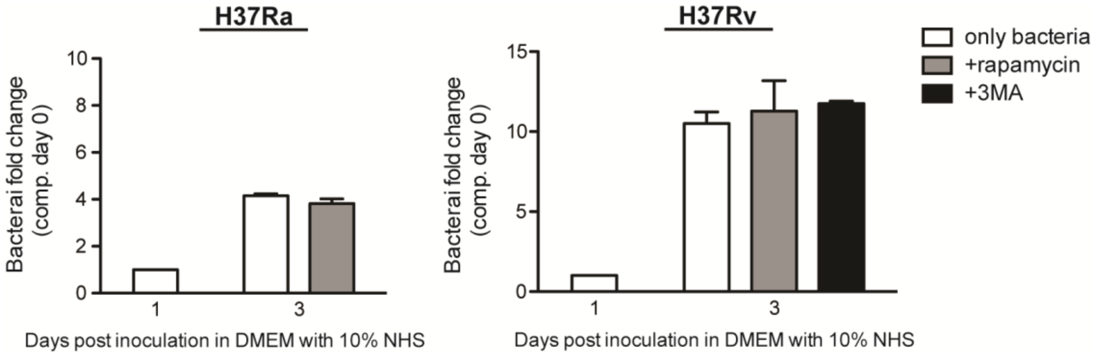

Supplementary Figure S1. Rapamycin or 3MA do not affect Mtb growth in absence of hMDMs. H37Ra or H37Rv was grown with or without continuous presence of 1μM rapamycin or 1mM 3MA and the bacterial growth was measured at the indicated days, showing no difference with or without inhibitors, in the absence of hMDMs. Data are presented as mean ± SEM of 3 independent experiments. (Related to Figure 2 in the main manuscript)

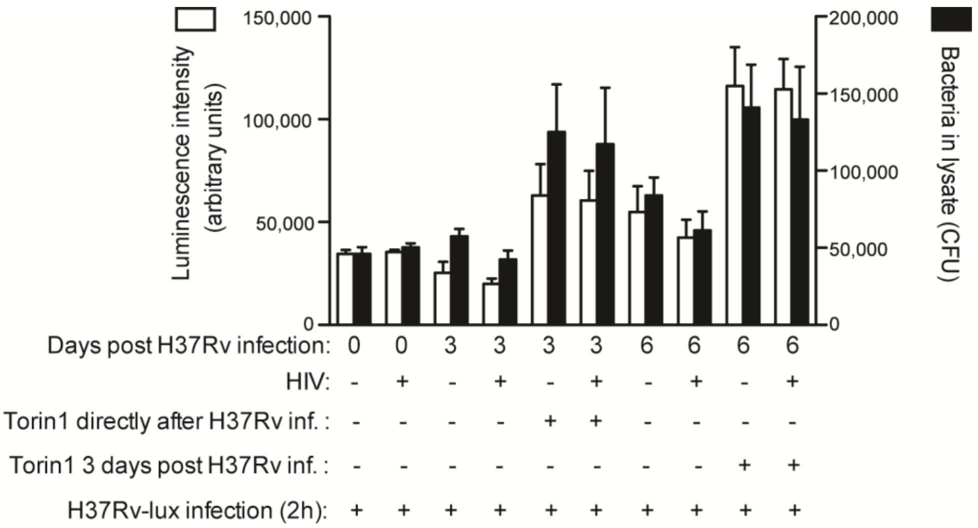

Supplementary Figure S2. CFU plating confirming the luminescence result of Mtb replication after different timing of autophagy induction. hMDMs were pre-infected with/without HIV for seven days before infected with H37Rv (MOI=1) for 2 hours. hMDMs were then incubated with/without Torin1 (250nM) for 3 days, either added directly or 3 days post infection. The graph shows the level of bacteria in cell lysates as measured by luminescence (from luciferase expressing Mtb; H37Rv-lux), or CFU plating performed in triplicates. Data are mean ± SEM from 4-5 independent experiments were luminescence and CFU was determined in the same lysates (same donors and experiments as analyzed in Figure 4 in the main manuscript).

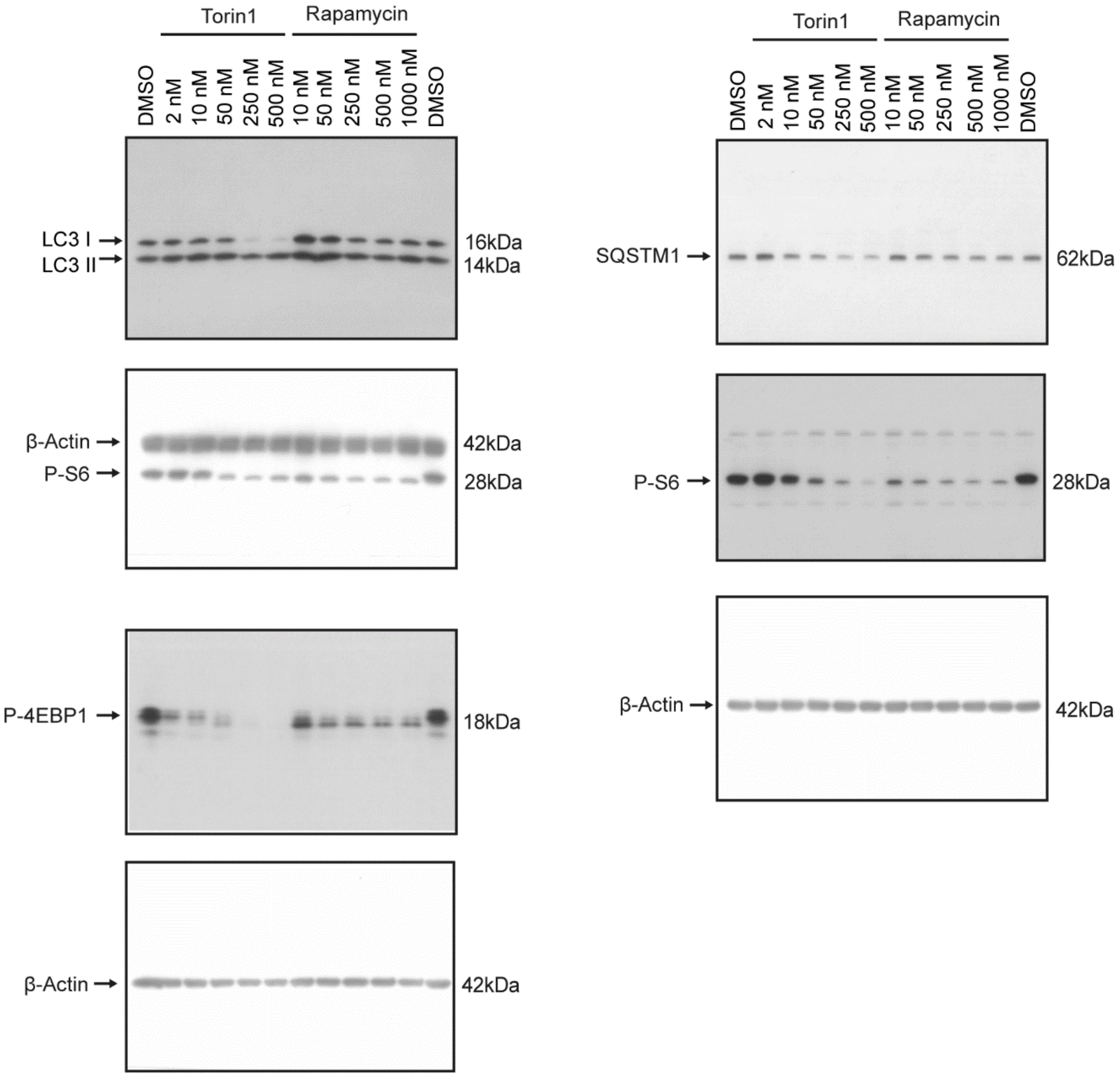

Supplementary Figure S3. The efficient autophagy inducer Torin1 causes a dose-dependent increase in Mtb growth in co-infected hMDMs. Full-length blots of Figure 3a, showing LC3, SQSTM1, P-S6 and P-4EBP1 with their respective β-actin that was run on the same gel. In the blot of β-actin to the upper left there is also a band of P-S6 since the membrane was probed for this protein prior to β-actin. Arrows point to the indicated proteins.

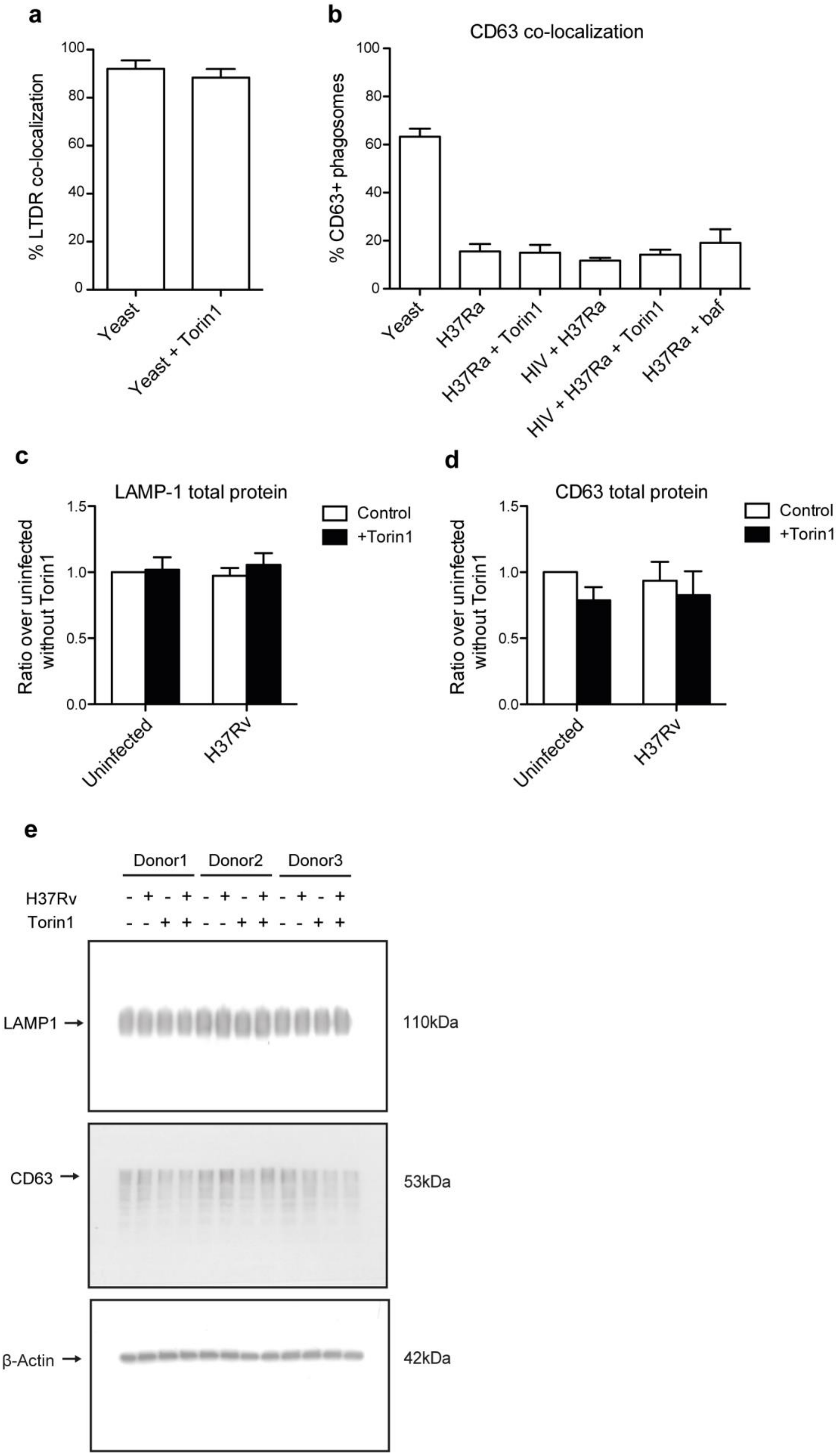

a
% LTDR co-localization
100
80
60
40
20
0
Yeast
Yeast + Torin1
b
CD63 co-localization
% CD63+ phagosomes
Yeast
H37Ra
H37Ra + Torin1
HIV + H37Ra
HIV + H37Ra + Torin1
H37Ra + baf
c
LAMP-1 total protein
Ratio over uninfected without Torin1
1.5
1.0
0.5
0.0
Control
+Torin1
Uninfected
H37Rv
d
CD63 total protein
Ratio over uninfected without Torin1
Control
+Torin1
Uninfected
H37Rv
e
Donor1
Donor2
Donor3
H37Rv
Torin1
LAMP1
110kDa
CD63
53kDa
β-Actin
42kDa

Supplementary Figure S4. Torin1 does not decrease acidification of yeast phagosomes and does not affect protein levels of LAMP-1 and CD63. (a) Percentage of LTDR co-localization with yeast (MOI=5) phagosomes 6h post infection, with Torin1 (250nM) added the last 4h. (b) Percentage of CD63 co-localization to yeast (MOI=5) or Mtb (MOI=1) phagosomes 6h post infection, with Torin1 (250nM) added the last 4h, in hMDMs pre-infected with/without HIV for 7 days. Bafilomycin (baf) was added 1h prior to Mtb infection. (c-d) The graphs shows the densitometry measurements of the total protein level of LAMP-1 resp. CD63 from western blots shown in (e). The values were normalized to their respective β-actin control and are presented as ratio over uninfected without Torin1. The data are shown as mean ± SEM of 3 independent experiments for (a), (c) and (d) and of 6 independent experiments for (b).

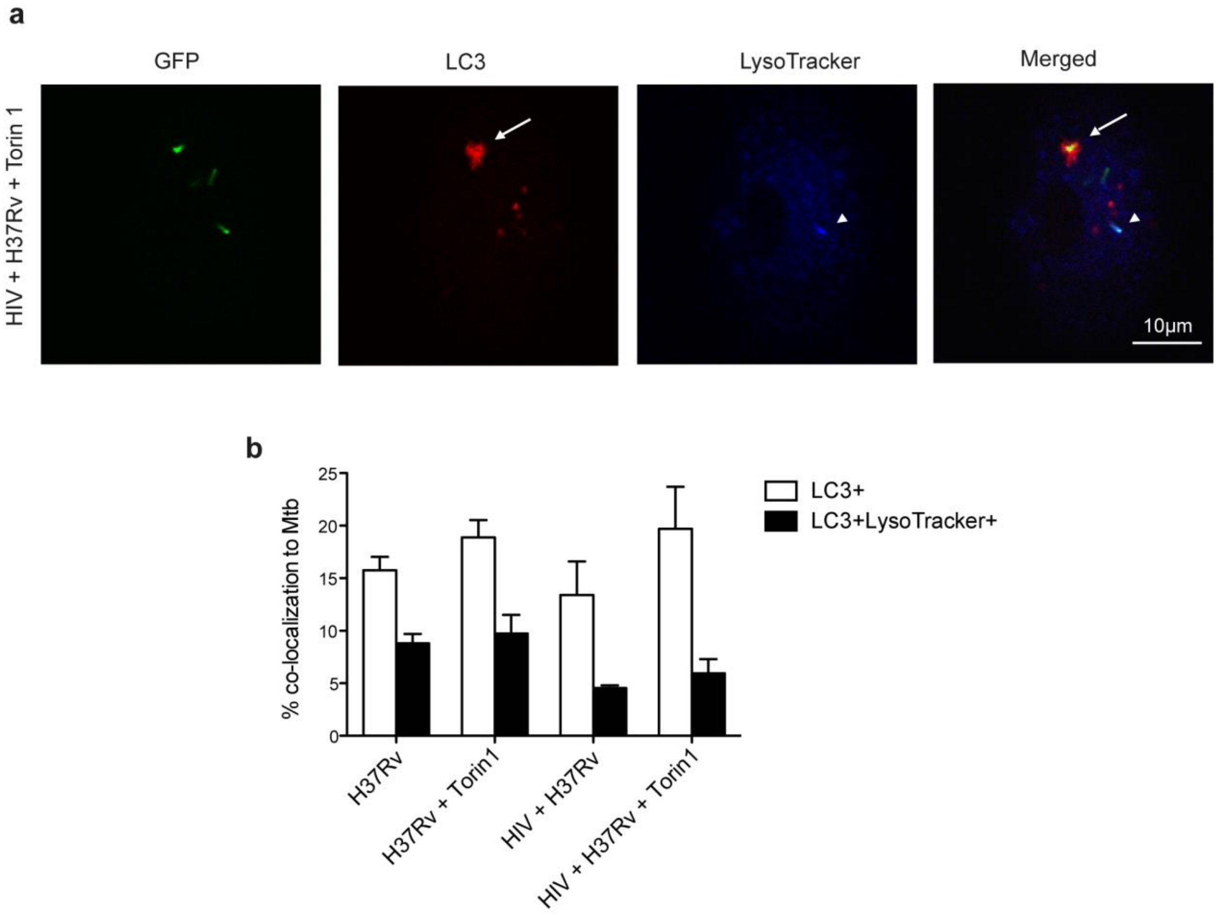

Supplementary Figure S5. Torin1 increases the number of autophagosomes without causing an increase in autophagolysosomes. hMDMs were pre-infected with HIV for 7 days before infection with H37Rv (MOI=1) for 6h, with the addition of Torin1 for the last 4h. (a) Representative micrographs of LysoTracker and LC3 co-localization to Mtb phagosomes in hMDMs co-infected with HIV/H37Rv and stimulated with Torin1. The arrowheads indicate co-localization to Mtb with LysoTracker, while the arrows point to LC3 positive phagosomes. Green: Mtb, red: LC3, blue: LysoTracker. (b) Percentage of LC3+ and LC3+LysoTracker+ phagosomes shown as mean ± SEM of 5 independent experiments for single infected cells and 2 independent experiments for co-infected cells.

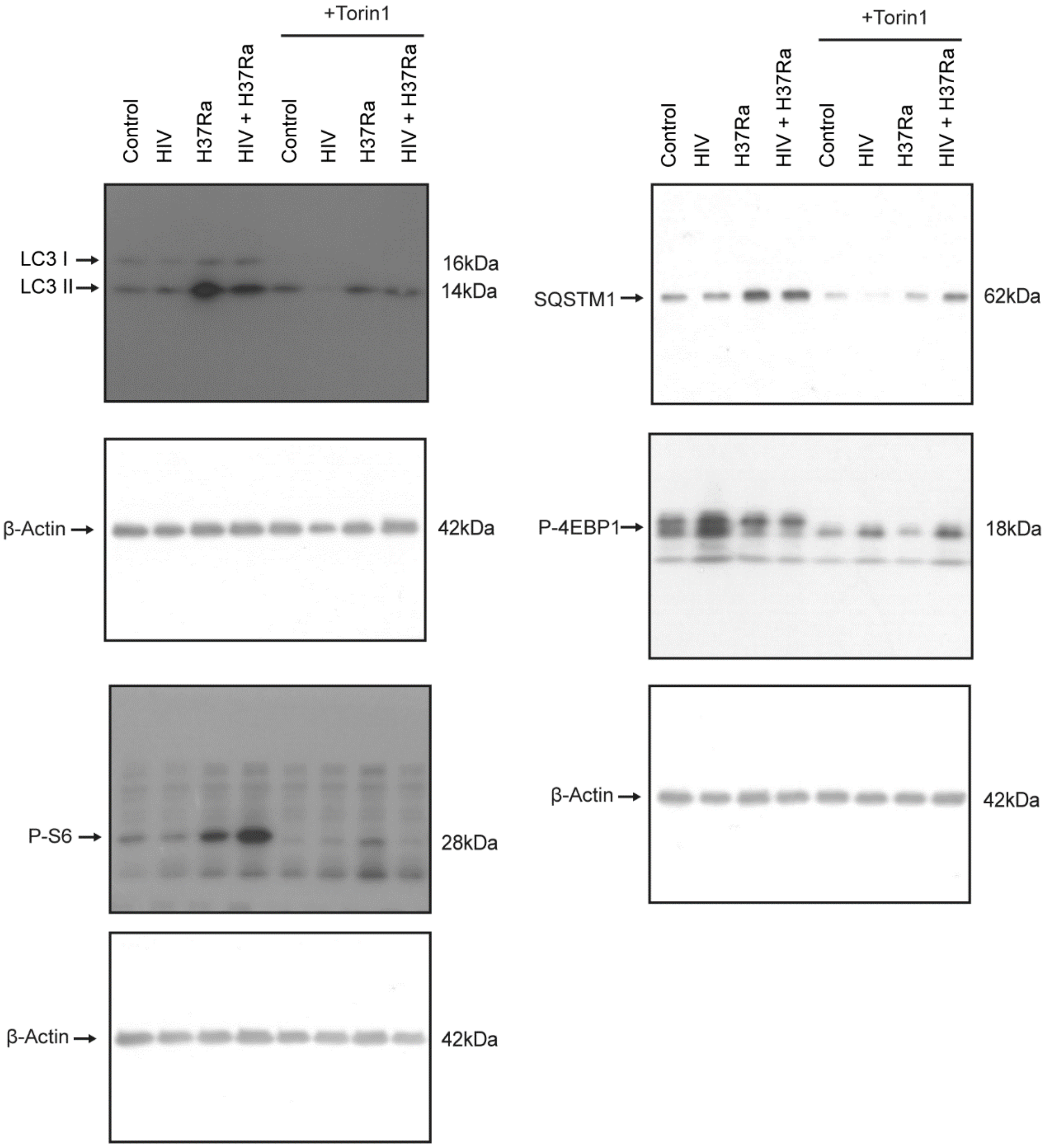

Supplementary Figure S6. Torin1-induced autophagy and flux is cellular and not localized to Mtb phagosomes. Full-length blots of Figure 7a in the main manuscript, showing LC3, SQSTM1, P-S6 and P-4EBP1 with their respective β-actin that was run on the same gel. Arrows point to the indicated proteins.

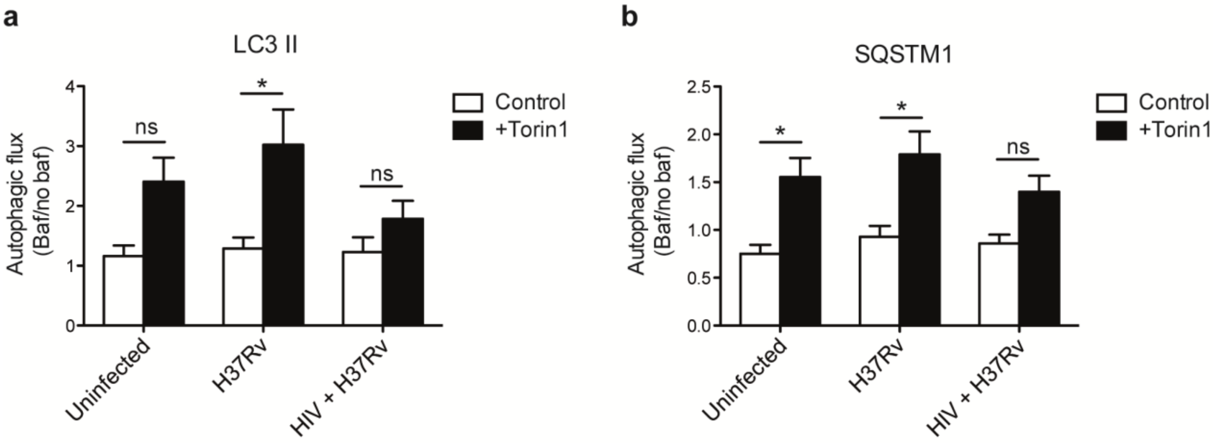

Supplementary Figure S7. Torin1 causes an autophagic flux on a cellular level, in Mtb infected but not in HIV co-infected hMDMs. hMDMs were pre-infected with HIV for 7 days prior to Mtb infection for 6h, with addition of Torin1 (250nM) the last 4h. Bafilomycin (baf) was added 1h prior to Mtb infection. The graphs show the densitometry measurements of the total protein level of LC3 II (a) and SQSTM1 (b) from western blot. The values are normalized to their respective β-actin control and are presented as ratio between bafilomycin treated cells and cells without bafilomycin treatment in order to study flux in control cells and Torin1 treated cells (>1 = flux, <1 = no flux). The data are shown as mean ± SEM with * $p<0.05$ using paired Student t-test of 5 independent experiments.

Primers for selected genes

The specific primers for the selected genes had the following sequences: *B2M*: 5´-TGGAGCATTCAGACTTGTCTTTC-3´ and 5´-ACACGGCAGGCATACTCAT-3´; *ACTB*: 5´-ACCCAGCACAATGAAGATCA-3´ and 5´-TCGTCATACTCCTGCTTGCT-3´; *GAPDH*: 5´-ACCAGGTGGTCTCCTCTGAC-3´ and 5´-TTGCTGTAGCCAAATTCGTT-3´; *ATG4A*: 5´-ACACTGGCCTCCCTTTGTAC-3´ and 5´-CCTCCAGATCAAACTCCTCCA-3´; *ATG5:* 5´-GGGCCATCAATCGGAAACTC-3´ and 5´-CCACAGGACGAAACAGCTTC-3´; *BECN1:*5´-GGAGCTGCCGTTATACTGTTC-3´ and 5´-TCTTTGAACTGCTGCACACA-3´; *MAP1LC3B*: 5´-AGTTCCTTGTACCTGACCATGT-3´ and 5´-GACCATGCTGTGTCCGTTC-3´; *ATG12*: 5´-GTGTTGCAGCTTCCTACTTCA-3´ and 5´-GGAAACTGCAGCGGAAGAC-3´; *ATG16L2*: 5´-TGTTCCCGAGACAACACACT-3´ and 5´-ACAGCTTTGGTCCAGTCAGA-3´; *SQSTM1*: 5´-CTGCCCAGACTACGACTTGT-3´ and 5´-GTGTCCGTGTTTCACCTTCC-3´.
